# Survival probability and predictors for woman experience childhood death in Nigeria: “analysis of north–south differentials”

**DOI:** 10.1186/1471-2458-12-430

**Published:** 2012-06-12

**Authors:** Ayo S Adebowale, Bidemi O Yusuf, Adeniyi F Fagbamigbe

**Affiliations:** 1 Department of Epidemiology, Medical Statistics and Environmental health, Faculty of Public Health, College of Medicine, University of Ibadan, Ibadan, Nigeria

**Keywords:** Childhood Mortality, Probability, Life table, Nigeria

## Abstract

**Background:**

Childhood mortality rate is high in Nigeria. There is dearth of information on the comparison of childhood mortality probability and its causal factors in the Northern and Southern Nigeria. This study was designed to fill these gaps.

**Methods:**

Nigeria Demographic and Health Survey, 2008 data was used. The first part of this study focused on women aged 15–49 who ever given birth to a child (n = 23,404), irrespective of the survival status of the child and the second part utilized all women aged 15–49 (N = 33,385). The outcome variable was experienced childhood mortality. Data was analyzed using Chi-square, logistic regression and Brass logit model.

**Results:**

Results showed that similar patterns of children’s death were observed in the two regions, but variation existed. Childhood mortality experienced was more pronounced in the North than the South, even when the potential confounding variables were used as control. Levels of education and wealth index showed an inverse relationship with childhood death in the regions (p < 0.05). The gap in childhood mortality experienced between the poorest and richest was wider in the North than the South. There was no significant difference in the risk of childhood mortality experienced by women in the urban and rural areas in the North (p > 0.05), but the difference was significant in the South (p < 0.05). The life-table mortality levels were lower in the North than the South, an indication of higher previous childhood mortality experience in the North than in the South. Across all childhood ages, the smoothed childhood mortality probabilities were consistently higher in the North than the South.

**Conclusion:**

Childhood mortality is higher in the Northern than Southern Nigeria. Improving women’s education, particularly in the North will alleviate childhood mortality in Nigeria.

## Background

There is global reduction in childhood mortality, but the trend differs between developed and developing countries [1]. The highest childhood mortality worldwide is found in sub-Saharan African region, where 1 child in 8 dies before age five, nearly 20 times the average of 1 in 167 for developed regions [2]. Nigeria, the most populous country in the region has a high rate of childhood mortality with the prevalent of 157 deaths per 1,000 births [3].

Despite high national estimate of childhood mortality in Nigeria, differential existed across the six geopolitical zones, where under-five mortality ranges from 89 deaths per 1,000 births in south west to 222 deaths per 1,000 births in north east [3]. Infant mortality is also lowest in south west (59 deaths per 1,000 births) and highest in north east (109 deaths per 1,000 births) [3]. The variation in childhood mortality is largely due to prevention strategies and socio-cultural differences across the country [3-7].

Previous studies conducted in Nigeria have shown that the north and south are different in some socio-economic conditions [3,4,7]. For instance, illiteracy level, polygamous marriage, early marriage (teenage pregnancy), poor utilization of modern health facility, proportion of rural residence and poverty are predominantly higher in the north than the south [2,4,8]. Each of these factors have been shown to have strong influence on childhood mortality in different countries and more significantly when a family exhibits more than one of the conditions [9-12]. However, the current study adds to existing knowledge on north south differential in experienced childhood deaths by women in Nigeria [8,13-16]. There is also dearth of information on the estimate of adjusted childhood mortality probabilities in the northern and southern Nigeria. Therefore, this study was designed to fill these gaps.

The objectives of this study were to; provide adjusted estimate of childhood mortality probabilities and life-table mortality levels for northern and southern parts of Nigeria, determine the prevalence of experienced childhood mortality in Nigeria and examine socio-demographic differential in experienced childhood mortality in the northern and southern Nigeria.

The first objective was designed with the view to knowing the level of mortality in the two regions. The childhood mortality probabilities were also adjusted to provide better estimate since demographic data from developing countries like Nigeria are characterized by incompleteness and data quality problems [15]. The vision to expunge the influence of poor data on the estimate of childhood mortality motivated the researchers to adopt the technique used in this study.

The experienced childhood mortality is necessary to evaluate the impact of the past and recent childhood mortality reduction strategies put in place by Nigerian governments and international agencies. This will also reveal which region in Nigeria has failed in actualization of these strategies. Studying the differential in experienced childhood mortality by women of childbearing age in the northern and southern Nigeria is indispensable, as this will also provide decisive information for planning and evaluating success of health services and interventions. It will aid the planners and policy makers in their decisions and support existing framework on childhood mortality reduction actions in Nigeria.

## Methods

Nigeria came into existence as a nation-state in 1914 through the amalgamation of the Northern and Southern protectorates. Nigeria now has six geo-political zones, these are; North East, North West, North Central, South East, South West and South South. These zones can equally be classified into North involving all the zones in the North and South combining all the zones in the South. Population Census held in 2006 puts Nigeria’s population at 140,431,790 million inhabitants, with a national growth rate estimated at 3.2 percent per annum and population doubling time of 22 years [7]. The population figure makes Nigeria the most populous nation in Africa [17]. The country is ravaged with poverty and also threatened by HIV/AIDS. The prevalent of HIV/AIDS in Nigeria ranks among the highest globally [17]. Poor attitudes of past governments in Nigeria towards health and other infrastructural programmes pose a serious challenge to survival chances of its citizens, particularly children.

### Data collection

#### Sample allocation and selection

This study which focused on women of childbearing age utilized data that were extracted from the survey conducted by ICF Macro Calverton, Maryland, USA in conjunction with National Population Commission (NPC), Nigeria (Nigeria Demographic and Health Survey, 2008 (NDHS). However, during the survey a stratified two-stage cluster design was used to select the eligible respondents. The primary sampling unit (PSU), a cluster for the survey was defined on the basis of Enumeration Areas (EAs) from the 2006 census frame. A minimum requirement of 80 households for the cluster size was used in the design.

Due to the need to obtain estimates for each of the 36 states in Nigeria and FCT-Abuja, the number of clusters in each state was not allocated proportional to their total population (or households). Based on the level of non-response found in the 2003 Nigeria DHS, approximately 36,800 households were selected, and all women aged 15-49 were interviewed. However, since the sample was unbalanced among residence area and state, a final weighing adjustment procedure to provide estimates at every other domain of study was used.

A well structured questionnaire was designed for the survey and the interviewers were trained. A pilot survey was conducted to check international consistency and reliability of the survey instruments at different locations before the actual study began. Questions on births and death history of children were included in the questionnaire. This paved way for the analysis methods adopted in the current study.

### Measurement scale for categorization of wealth index

For the computation of wealth index, principal components analysis (PCA) was used to assign the wealth indicator weights. This procedure firstly assigned scores and standardized the wealth indicator variables such as; bicycle, cars, building type, e.t.c. Thereafter, the factor coefficient scores (factor loadings) and z-scores were calculated. Finally, for each household, the indicator values were multiplied by the loadings and summed to produce the household’s wealth index value. The standardized z-score was used to disentangle the overall assigned scores to poorest, poorer, middle, richer, richest categories [3].

### Data analysis

In the first part of the study, the outcome variable is whether a woman had experienced at least one childhood death previously. The analysis began with cross tabulation of the outcome variable (under-five mortality) and selected socio-demographic factors. Chi-square (χ^2^) test was used to determine if there are associations between some socio-demographic variables and child mortality. Thereafter, variables found to be significant in the analysis were entered into a logistic regression model to predict the strength of the associations between these variables and childhood mortality. All analyses were carried out at 5% level of significant. The variable childhood mortality experienced was dichotomized into two by creating a dummy variable 0 (zero) for those who had not experienced childhood deaths and 1(one) if otherwise. Here a reference category (Ref.) was created which was used as bases of comparison between subgroup of the study sample.

The logistic regression model is defined as;

(1) $$log\left(\frac{p}{1-p}\right)=\alpha_{0}+\beta_{1}x_{1}+\beta_{2}x_{2}+\beta_{3}x_{3}+\ldots +\beta_{k}x_{k}$$

Where; p is the proportion of women who had experienced childhood deaths. $\beta_{1},\beta_{2},\beta ,\ldots ,\beta_{k}$ , are the regression coefficients to be estimated; $x_{1},x_{2},x_{3},\ldots ,x_{k}$ are covariates such as age, level of education, religion, residence and wealth index.

In the second part of this study, childhood survivorship probabilities and life-table mortality level were estimated using indirect method that was first developed by William Brass in late 1960’s [18] which was later modified by Coale and Trussel [19]. The approach has been widely used for the analysis of childhood mortality in different countries and the outcome had produced the expected results [16,20].

The data required are; age of the mother and fertility experience of true cohorts. That is, data on children ever born and children surviving. These were used to estimate the number of children dead. Average parity per woman P(i) and proportion of children dead D(i) for each age group of mother were computed.

For adjustment, multiplier K(*i*) was used i.e

(2) K(i)=a(i)+b(i)[P(1)/P(2)+c(i)[P(2)/P(3)]

was estimated for each age group of women.

Where, a(*i*), b(*i*) and c(*i*) are constant coefficients and were chosen from the North model life table [21]. Thereafter, probabilities of dying q(x) and probabilities of surviving *l*(x) were determined i.e. q(x)=K(i)D(i); The reference dates were obtained using the model;

(3) t(i)=A(i)+B(i)[P(1)/P(2)]+C(i)[P(2)/P(3)]

Where, A(*i*), B(*i*) and C(*i*) are constant coefficients and were chosen from the North model life table. Since the NDHS survey was conducted between June and October 2008, the period was put at 2008.8. The t(i) values were therefore subtracted from this date to give the reference date.

Mortality level at each age was estimated using the Coale-Demeny system. The probabilities of surviving *l*_*x*_*(i)* were smoothed using Brass logit system of equations and with assumption of one-parameter model [20]. These equations are;

(4) $$\text{logit}\{l(\text{z)}\}=\frac{1}{2}\log_{\text{e}}\left\{\frac{l(\text{z})}{1-l(\text{z})}\right\}$$

(5) $$\log\text{it}\{l_{x}(i)\}=\alpha +\text{β}\log it\left\{l_{x}(\text{s})\right\}$$

Estimate of ($\hat{\alpha }$) was obtained from these equations using the average of the mortality levels of *l*_*1*_(*i*), *l*_*2*_(*i*), *l*_*3*_(*i*) and *l*_*5*_(*i*) from the index and *l*_*1*_(*s*), *l*_*2*_(*s*), *l*_*3*_(*s*) and *l*_*5*_(*s*) from the standard population.

For the Northern Nigeria;

(6) $$\text{Average early childhood mortality level=}\frac{M_{N}L(1)+M_{N}L(2)+M_{N}L(3)+M_{N}L(5)}{4}$$

For the Southern Nigeria;

(7) $$\text{Average early childhood mortality level=}\frac{M_{S}L(1)+M_{S}L(2)+M_{S}L(3)+M_{S}L(5)}{4}$$

The average mortality level for each of the regions was used to select the appropriate Coale-Demeney Model life table. Thereafter, equation (2) was reduced to 1-parameter model by equating β to 1. Thus provided means of estimating ∝ i.e $\hat{\alpha }={\overset{―}{Y}}_{x}(i)-{\overset{―}{Y}}_{x}(s)$.

Where; ${\overset{―}{Y}}_{x}(i)=\overset{―}{\log\text{it}\{l_{x}(i)\}}\text{and}{\overset{―}{Y}}_{x}(s)=\overset{―}{\log\text{it}\{l_{x}(s)\}}$

Therefore; $\hat{\alpha }={\overset{―}{Y}}_{x}(i)-{\overset{―}{Y}}_{x}(s)$

(8) $$=\frac{Y_{2}(i)+Y_{3}(i)+Y_{5}(i)}{3}-\frac{Y_{2}(i)+Y_{3}(s)+Y_{5}(s)}{3}$$

$\hat{\alpha }$ was estimated separately for Northern and Southern Nigeria.

### Ethical considerations

The ethical approval was obtained from Nigeria National Ethics Committee (RNEC) functioning under the Ministry of Health, Nigeria. An informed consent was obtained from all the study participants after describing to them all the issues related to the study in details at the point of data collection. Eligible respondents who did not want to partake in the study were excluded from the survey. Each consented participants was made to sign appropriate agreement form before the interview.

### Study limitations

The major limitation is that some of the women who had experienced childhood mortality might have claimed that they never experienced such. They see this as event that one should not remember again. This may bias the number of women who had experienced childhood mortality in Nigeria.

## Results

### Background characteristics of the study population

Table 1 shows the Chi-square distribution of selected background characteristics of women aged 15–49 by northern and southern regions in Nigeria, 2008. The data was evidenced that all the background characteristics (age, residence, education, wealth index and religion) were significantly associated with region (northern and southern) (p < 0.001). Also, differential existed across subgroup of women in the two regions. The data further depict that the youngest age group (15–19) consisted of 6.8% and 2.6% of the total sample from the northern and southern Nigeria respectively. Across the two regions, majority of the respondents were between the ages of 25–29 (North (21.5%) and South (21.7%)) and higher number of respondents was observed in the rural than urban areas. Approximately, 68% of the respondents were living in the rural area and rural residents in the north constituted higher percentage (78.1%) of the respondents from the north, while it constituted 55.2% in the south.

**Table 1:** Distribution of Selected Background Characteristics of Women aged 15–49 by Northern and Southern Regions in Nigeria, 2008

**Background Characteristics**	**Region**		**Total**	**χ**^**2**^**-value**	**p-value**
	**North**	**South**			
**Total**	**100.0(13359)**	**100.0(10047)**	**100.0(23406)**		
**Age**				**445.292**	**p < 0.001**
15-19	6.8(908)	2.6(263)	5.0(1171)		
20-24	17.5(2336)	11.5(1154)	14.9(3490)		
25-29	21.5(2875)	21.7(2176)	21.6(5051)		
30-34	17.0 (2274)	19.6(1967)	18.1(4241)		
35-39	14.2(1897)	18.2(1826)	15.9(3723)		
40-44	11.8(1575)	13.5(1356)	12.5(2931)		
45-49	11.2(1494)	13.0(1305)	12.0(2799)		
**Mean Age**	**31.2 ± 8.8**	**36.6 ± 7.9**	**33.4 ± 8.2**		
**Residence**				**1381.114**	**p < 0.001**
Urban	21.9(2927)	44.8(4496)	31.7 (7423)		
Rural	78.1(10430)	55.2(5550)	68.3(15980)		
**Education**				**7772.909**	**p < 0.001**
None	69.4 (9266)	12.8(1283)	45.1 (10549)		
Primary	16.8(2249)	31.7 (3180)	23.2(5429)		
Secondary	10.8(1449)	43.5 (4372)	24.9(5821)		
Higher	2.9(393)	12.1(1211)	6.9(1604)		
**Wealth Index**				**5951.738**	**p < 0.001**
Poorest	34.3 (4580)	5.5(550)	21.9(5130)		
Poorer	28.0(3737)	12.2(1222)	21.2(4959)		
Middle	18.7(2498)	19.8(1994)	19.2(4492)		
Richer	12.0(1601)	27.3 (2744)	18.6(4345)		
Richest	7.1(942)	35.2(3536)	19.1(4478)		
**Religion**				**8082.735**	**p < 0.001**
Christian	21.8(2910)	80.3 (8071)	46.9 (10981)		
Islam	75.9(10140)	17.3(1739)	50.8 (11879)		
Traditional	1.4(191)	1.8(181)	1.6 (372)		
Others	0.9(117)	0.5(55)	0.7(172)		

According to education, majority of the women had no education (45.1%). These sets of women were mostly common in the north (69.4%) than the south (12.8%). Highest proportion of women in the north had no education (69.4%), whereas in the south, majority had secondary education (43.5%) respectively. With respect to wealth index, 34.3% of women in the north belonged to poorest wealth index as against only 5.5% from the south. Majority of the women in the south (35.2%) were in the richest wealth index category. The most prominent religion of women in the north was Islam (75.9%), while in the south, majority of the women were Christians (80.3%). However, very few respondents were traditional religion followers (North (1.4%) vs South (1.8%)).

Table 2 depicts the Chi-square distribution of childhood mortality experience by women aged 15–49 in the northern and southern Nigeria. The data show that there was a significance association between childhood mortality experience and region (p < 0.001). Across the two regions, lower percentages of women had experienced childhood mortality. The prevalence of childhood mortality experienced in Nigeria was 40.3%, with women in the north (47.3%) ever experienced higher childhood mortality than their counterparts in the south (30.9%).

**Table 2:** Distribution of Childhood Mortality Experience by Women of aged 15–49 in Northern and Southern, Nigeria, 2008

**Region**	**Child Mortality Experience**		**χ**^**2**^**-value**	**p-value**	
	**No**	**Yes**	**Total**	**644.802**	**p < 0.001**
North	52.7(7035)	47.3(6323)	100.0(13358)		
South	69.1(6943)	30.9(3103)	100.0(10046)		
**Total**	**59.7(13978)**	**40.3(9426)**	**100.0(23404)**		

Table 3 shows the percentage distribution of children according to child survival status by selected background characteristics of mothers in the Northern and Southern Nigeria. The mean ages of respondents in the Northern and Southern Nigeria were 31.2 ± 8.8 and 33.4 ± 8.2 respectively. Those who had experienced childhood death were older in age than those who had not. The data revealed that similar patterns of death among children were observed in the Northern and Southern regions across the selected socio-demographic characteristics. Variation existed in these patterns across subgroup of women in the two regions. However, experienced childhood mortality was more pronounced in the North (47.3%) than the South (30.1%). Significant associations existed between children survival status and age (p < 0.001), place of residence (p < 0.001), education (p < 0.001), wealth index (p < 0.001) and religion (p < 0.001).

**Table 3:** Distribution of Childhood Mortality Experience by Selected Background Characteristics of Women of aged 15–49 in Northern and Southern, Nigeria, 2008

**Background**	**North**			**South**		
	**Child Mortality**			**Child Mortality**		
**Characteristics**	**No**	**Yes**	**Total**	**No**	**Yes**	**Total**
Total	7034(52.7)	6324(47.3)	100.0(13358)	69.1(6944)	30.1(3102)	100.0(10046)
**Current Age**	**p < 0.001; χ**^**2**^**-value = 1417.224**			**p < 0.001; χ**^**2**^**-value = 691.626**		
15-19	83.9(761)	16.1(146)	100.0(907)	92.4(243)	7.6(20)	100.0(263)
20-24	71.4(1669)	28.6(667)	100.0(2336)	85.4(986)	14.6(168)	100.0(1154)
25-29	60.7(1745)	39.3(1130)	100.0(2875)	77.9(1695)	22.1(481)	100.0(2176)
30-34	49.0(1114)	51.0(1160)	100.0(2274)	73.8(1451)	26.2(516)	100.0(1967)
35-39	39.6(752)	60.4(1145)	100.0(1897)	63.1(1152)	36.9(674)	100.0(1826)
40-44	34.9(549)	65.1(1026)	100.0(1575)	58.7(796)	41.3(560)	100.0(1356)
45-49	29.7(444)	70.3(1050)	100.0(1494)	47.6(621)	52.4(683)	100.0(1304)
**Mean Age**	**28.5 ± 8.2**	**34.2 ± 8.5**	**31.2 ± 8.8**	**32.0 ± 7.9**	**36.6 ± 7.9**	**33.4 ± 8.2**
**Residence**	**p < 0.001; χ**^**2**^**-value = 83.361**			**p < 0.001; χ**^**2**^**-value = 142.934**		
Urban	60.1(1760)	39.9(1168)	100.0(2928)	75.2(3383)	24.8(1113)	100.0(4496)
Rural	50.6(5275)	49.4(5155)	100.0(10430)	64.2(3561)	35.8(1989)	100.0(5550)
**Education**	**p < 0.001; χ**^**2**^**-value = 480.136**			**p < 0.001; χ**^**2**^**-value = 406.498**		
None	47.4(4394)	52.6(4872)	100.0(9266)	57.2(735)	42.8(549)	100.0(1284)
Primary	56.1(1262)	43.9(987)	100.0(2249)	60.0(1908)	40.0(1271)	100.0(3179)
Secondary	73.6(1067)	26.4(383)	100.0(1450)	75.1(3283)	24.9(1089)	100.0(4372)
Higher	79.4(312)	20.6(81)	100.0(393)	84.0(1017)	16.0(194)	100.0(1211)
**Wealth Index**	**p < 0.001; χ**^**2**^**-value = 289.803**			**p < 0.001; χ**^**2**^**-value = 272.048**		
Poorest	47.9(2195)	52.1(2385)	100.0(4580)	64.5(355)	35.5(195)	100.0(550)
Poorer	48.2(1801)	51.8(1936)	100.0(3737)	60.7(742)	39.3(480)	100.0(1222)
Middle	54.5(1361)	45.5(1137)	100.0(2498)	60.0(1196)	40.0(797)	100.0(1993)
Richer	61.6(987)	38.4(614)	100.0(1601)	68.2(1872)	31.8(872)	100.0(2744)
Richest	73.5(692)	26.5(250)	100.0(942)	78.6(2778)	21.4(758)	100.0(3536)
**Religion**	**p < 0.001; χ**^**2**^**-value = 141.423**			**p < 0.001; χ**^**2**^**-value = 49.805**		
Christian	61.6(1793)	38.4(1116)	100.0(2909)	68.6(5533)	31.4(2538)	100.0(8071)
Islam	50.0(5067)	50.0(5073)	100.0(10140)	73.8(1283)	26.2(456)	100.0(1739)
Traditional	65.4(125)	34.6(66)	100.0(191)	50.0(90)	50.0(90)	100.0(180)
Others	41.9(49)	58.1(68)	100.0(117)	67.3(37)	32.7(18)	100.0(55)

Across all age groups, the percentage of women having at least a dead child increases with increasing in age of women, but higher childhood deaths experience was recorded by women in the North than their counterparts in the South. In the North, there was an increase from 16.1% among women in age group 15-19 yrs to 70.3% among women in age group 45–49, whereas in the South, the increase was from 7.6% to 52.4% among women in age group 15–19 and 45–49, respectively. In either of the regions, rural women experienced higher proportion of childhood mortality than urban women, but the prevalent was higher among rural women in the North (49.4%) than those women living in the rural areas in the South (35.8%).

The percentage of mothers that have lost at least a child in the past fell consistently as the levels of education increases but at pal in the two regions. However, across all the education categories, women living in the North experienced higher childhood mortality than those in the South. The percentage of Christian mothers in the North (38.4%) who experienced childhood mortality was higher than Southern Christian mothers (31.4%). Striking differentials existed between childhood mortality experience of mothers who belong to Islamic religious sects in the North (50.0%) and in the South (26.2%). Meanwhile, children of mothers who are traditional followers in the North (34.6%) had lower childhood mortality than their counterparts in the South (50.0%).

Wealth index also showed an inverse relationship with experienced childhood deaths in the two regions. The gap in childhood mortality between the poorest and richest was wider in the North than the South. It is alarming to know that the percentage of mothers who had lost at least a child in the poorest wealth category in the South (35.5%) was less than that of the richer in the North (38.4%)

The unadjusted and adjusted logistic regression of relationship between childhood mortality experience of women aged 15–49 in Northern and Southern Nigeria are shown in Table 4. The data is an evidence of clear significant differential in childhood mortality experience in the Northern and Southern Nigeria, even when potential confounding variables were used as control. Unadjusted logistic regression show that women residing in the North were more likely (OR = 2.01; C.I = 1.905-2.124) to have experienced childhood mortality than their counterparts in the South. However, the strength of the relationship was reduced (AOR = 1.44; C.I = 1.317-1.544) when age, residence, education, wealth index and religion were used as control.

**Table 4:** Unadjusted and Adjusted Logistic Regression of Relationship between Childhood Mortality Experience of Women Aged 15–49 and Background Characteristics

**Background Characteristics**	**UNADJUSTED**				**ADJUSTED**			
	**β**	**Exp(β)**	**95% C.I for Exp(β)**		**β**	**Exp(β)**	**95% C.I for Exp(β)**	
			**Lower**	**Upper**			**Lower**	**Upper**
**Region**								
North	0.699	2.011*	1.905	2.124	0.355	1.426*	1.317	1.544
South	(Ref.)	1.000	(Ref.)	(Ref.)	(Ref.)	1.000	(Ref.)	(Ref.)
**Age**								
15-19					−2.633	0.072*	0.060	0.087
20-24					−1.805	0.164*	0.146	0.185
25-29					−1.257	0.284*	0.257	0.315
30-34					−0.838	0.433*	0.390	0.480
35-39					−0.424	0.655*	0.589	0.728
40-44					−0.246	0.782*	0.699	0.874
45-49					(Ref.)	1.000	(Ref.)	(Ref.)
**Residence**								
Urban					−0.135	0.874**	0.809	0.944
Rural					(Ref.)	1.000	(Ref.)	(Ref.)
**Education**								
None					1.099	3.000*	2.545	3.535
Primary					0.988	2.686*	2.299	3.137
Secondary					0.620	1.860*	1.599	2.163
Higher					(Ref.)	1.000	(Ref.)	(Ref.)
**Wealth Index**								
Poorer					0.629	1.876*	1.645	2.139
Poor					0.647	1.909*	1.684	2.165
Middle					0.534	1.706*	1.519	1.915
Richer					0.376	1.456*	1.310	1.619
Richest					(Ref.)	1.000	(Ref.)	(Ref.)
**Religion**								
Islam					0.146	1.157*	1.071	1.251
Traditional					−0.163	0.849	0.677	1.066
Christianity					(Ref.)	1.000	(Ref.)	(Ref.)

*Significant at 0.1%; Ref. Reference Category; β is the regression coefficient to be estimated; exp(β) represents the odd of childhood mortality experience; C.I is the confidence interval which is the interval at which the true estimate of exp(β) falls.

Table 5 shows the result from the logistic regression analysis of relationship between child survival and selected background characteristics of women in Northern and Southern Nigeria. In the North, the data revealed that the higher the age of a woman, the higher the risk of mortality among children. There is no significant difference in the risk of childhood mortality in the urban and rural areas in the North (p > 0.05). Mothers who had no education, primary education and secondary education were 2.8(p < 0.001), 2.6(p < 0.001) and 1.7(p < 0.001) respectively more likely to have experienced childhood mortality than those with higher levels of education. Women who belong to traditional religion affiliations were less likely (0.607, p < 0.01) to have experienced childhood mortality than Christian women. Children of the women in the poorest, poorer, middle and richer wealth indices were 2.2(p < 0.001), 2.28(p < 0.001), 1.85(p < 0.001) and 1.47(p < 0.001) times more likely to die than those in the richest wealth category.

**Table 5:** Logistic Regression of Relationship between Childhood Mortality Experience and Selected Background Characteristics of Women Aged 15–49 in Northern and Southern Nigeria

**Background**	**North**				**South**			
	**95% C.I for Exp(β)**				**95% C.I for Exp(β)**			
**Characteristics**	**β**	**Exp(β)**	**Lower**	**Upper**	**β**	**Exp(β)**	**Lower**	**Upper**
**Current Age**								
15-19 (Ref.)	(Ref.)	1.000	(Ref.)	(Ref.)	(Ref.)	1.000	(Ref.)	(Ref.)
20-24	0.835	2.304*	1.886	2.815	0.803	2.232**	1.374	3.625
25-29	1.375	3.953*	3.255	4.801	1.400	4.053*	2.538	6.474
30-34	1.873	6.509*	5.340	7.934	1.664	5.280*	3.304	8.436
35-39	2.270	9.680*	7.903	11.858	2.129	8.407*	5.266	13.421
40-44	2.483	11.975*	9.714	14.762	2.291	9.883*	6.170	15.830
45-49	2.634	13.927*	11.256	17.232	2.648	14.127*	8.808	22.657
**Residence**								
Urban	(Ref.)	1.000	(Ref.)	(Ref.)	(Ref.)	1.000	(Ref.)	(Ref.)
Rural	0.053	1.054	0.945	1.176	0.200	1.222*	1.094	1.365
**Education**								
None	1.042	2.834*	2.133	3.766	0.973	2.647*	2.127	3.293
Primary	0.953	2.593*	1.952	3.444	1.036	2.818*	2.340	3.393
Secondary	0.510	1.665*	1.250	2.217	0.679	1.971*	1.652	2.353
Higher	(Ref.)	1.000	(Ref.)	(Ref.)	(Ref.)	1.000	(Ref.)	(Ref.)
**Wealth Index**								
Poorest	0.804	2.234*	1.822	2.738	0.256	1.292***	1.027	1.626
Poorer	0.825	2.282*	1.864	2.792	0.438	1.550*	1.295	1.854
Middle	0.613	1.846*	1.516	2.248	0.492	1.636*	1.407	1.902
Richer	0.385	1.469*	1.208	1.786	0.347	1.415*	1.244	1.610
Richest	(Ref.)	1.000	(Ref.)	(Ref.)	(Ref.)	1.000	(Ref.)	(Ref.)
**Religion**								
Christian	(Ref.)	1.000	(Ref.)	(Ref.)	(Ref.)	1.000	(Ref.)	(Ref.)
Islam	0.384	1.468*	1.323	1.629	−0.310	0.734*	0.646	0.833
Traditional	−0.500	0.607**	0.435	0.845	0.391	1.478***	1.078	2.027
Others	0.613	1.846**	1.226	2.779	−0.127	0.881	0.482	1.611

*Significant at 0.1%; **Significant at 1%; ***Significant at 5%; Ref. Reference Category; β is the regression coefficient to be estimated; exp(β) represents the odd of childhood mortality experience; C.I is the confidence interval which is the interval at which the true estimate of exp(β) falls.

In the South, the data revealed that the higher the age of a women, the higher the risk of mortality among children. Rural women were more likely to have experienced child death than urban women (OR = 1.2, p < 0.001). Mothers who had no education, primary education and secondary education were 2.65(p < 0.001), 2.82(p < 0.001) and 1.97(p < 0.001) times respectively more likely to have experienced childhood mortality than those with higher levels of education. Moreover, women who belong to traditional religion affiliations were more likely 1.48(p < 0.05) to have experienced childhood mortality than Christian mothers. However, childhood mortality was more pronounced among the Christians in the South than the Muslims.

Tables 6 and 7 show the derivation of probability of dying, smoothed probability of dying, mortality level and smoothed mortality level in Northern and Southern Nigeria. The probabilities of dying increased with the age of children in the two regions. Across ages 1,2,3,5,10,15 and 20 of the children, probabilities of dying were consistently higher in the North than the South, ranging from 0.1122 for infant to 0.2942 for children under age 20 in the North and 0.0769 for infant to 0.1662 for children under age 20 in the south. The life-table mortality levels were lower in the North than the South, showing an indication of higher previous childhood mortality experience in the North than in the South.

**Table 6:** Derivation of Probability of Dying, Mortality Levels and Reference Period, Northern Nigeria, NDHS 2008

**Age**	**x**	**FP(i)**	**CEB(i)**	**CA(i)**	**CD(i)**	**P(i)**	**D(i)**	**K(i)**	**q**_**x**_**(i)**	***l***_**x**_**(i)**	**ML**	**Ref. P**
*1*	*2*	*3*	*4*	*5*	*6*	*7*	*8*	*9*	*10*	*11*	*12*	*13*
15-19	1	3193	1209	1056	153	0.3786	0.1266	0.8864	0.1122	0.8879	13.26	2007.5
20-24	2	3169	5257	4350	907	1.6589	0.1725	0.9395	0.1621	0.8379	12.10	2006.1
25-29	3	3200	10199	8429	1770	3.1872	0.1736	0.9296	0.1614	0.8386	13.29	2004.3
30-34	5	2361	11377	8988	2389	4.8187	0.2100	0.9795	0.2057	0.7943	12.42	2002.2
35-39	10	1947	12043	9246	2797	6.1854	0.2323	1.0483	0.2435	0.7565	12.21	1999.9
40-44	15	1620	10731	8465	2266	6.6241	0.2112	1.0364	0.2189	0.7811	13.76	1997.4
45-49	20	1541	11771	8365	3406	7.6386	0.2894	1.0161	0.2941	0.7059	11.68	1994.6

FP(i): Female population; CEB(i): Children ever born; CA(i): Children alive; CD(i): Children dead; P(i): Average parity; D(i): Proportion of children dead; K(i): Multiplier; q_x_(i): Probability of dying; l_x_(i): Probability of surviving; ML: Mortality level; Ref. P: Reference period.

**Table 7:** Derivation of Probability of Dying, Mortality Levels and Reference Period, Southern Nigeria, NDHS 2008

**Age**	**x**	**FP(i)**	**CEB(i)**	**CA(i)**	**CD(i)**	**P(i)**	**D(i)**	**K(i)**	**q**_**x**_**(i)**	***l***_**x**_**(i)**	**ML**	**Ref. P**
*1*	*2*	*3*	*4*	*5*	*6*	*7*	*8*	*9*	*10*	*11*	*12*	*13*
15-19	1	3300	299	276	23	0.0906	0.0769	1.0536	0.0769	0.9231	16.55	2007.7
20-24	2	2964	2057	1839	218	0.6940	0.1060	1.0447	0.1060	0.8940	15.74	2006.7
25-29	3	3109	5662	5045	617	1.8212	0.1090	0.9974	0.1090	0.8910	16.36	2005.2
30-34	5	2273	6904	6140	764	3.0374	0.1107	1.0287	0.1107	0.8893	17.17	2003.4
35-39	10	1965	8537	7413	1124	4.3445	0.1317	1.0891	0.1317	0.8683	16.98	2001.3
40-44	15	1412	7335	6385	950	5.1948	0.1295	1.0712	0.1295	0.8705	17.56	1998.9
45-49	20	1332	7944	6624	1320	5.9640	0.1662	1.0465	0.1662	0.8338	16.51	1996.1

FP(i): Female population; CEB(i): Children ever born; CA(i): Children alive; CD(i): Children dead; P(i): Average parity; D(i): Proportion of children dead; K(i): Multiplier; q_x_(i): Probability of dying; l_x_(i): Probability of surviving; ML: Mortality level; Ref. P: Reference period.

The smoothening of mortality probabilities either reduces or decreases the unadjusted mortality probabilities in the two regions. In the North, the smoothed mortality probabilities were 0.1202, 0.15371, 0.17414, 0.20115, 0.23266, 0.24906 and 0.26740 for children aged 1, 2, 3, 5, 10, 15 and 20 respectively. Similar pattern but lower probabilities were obtained in the South. The reference period for these childhoods were also estimated to know when in the past can the mortality experienced be referred. Tables 8 and 9 show the procedures for estimating smoothed childhood probabilities of dying for Southern and Northern Nigeria.

**Table 8:** Derivation of Smoothed Probability of Surviving and Smoothed Mortality Levels for Northern Nigeria

**Age**	**x**	**l_x_(i)**	**Probabilities of surviving l_S_(i)**			**Logit {l_x_(i)}**	**Logit {l_x_(s)}**	**Logit {l_x_(l)**	**Smoothed Probability of Surviving**	**Smoothed Probability of Dying**
			**Level 12**	**Level 13**	**Level 12.77**					
*1*	*2*	*3*	*4*	*5*	*6*	*7*	*8*	*9*	*10*	*11*
15-19	1	0.8879	0.87255	0.88497	0.88211	−1.03473	−1.00628	−0.99529	0.87980	0.12020
20-24	2	0.8379	0.83620	0.85299	0.84913	−0.82134	−0.86390	−0.85291	0.84629	0.15371
25-29	3	0.8386	0.81397	0.83342	0.82900	−0.82392	−0.78928	−0.77829	0.82586	0.17414
30-34	5	0.7943	0.78466	0.80764	0.80236	−0.67552	−0.70056	−0.68957	0.79885	0.20115
35-39	10	0.7565	0.75097	0.77729	0.77124	−0.56679	−0.60766	−0.59667	0.76734	0.23266
40-44	15	0.7811	0.73375	0.76138	0.75503	−0.63604	−0.56281	−0.55182	0.75094	0.24906
45-49	20	0.7059	0.71487	0.74346	0.73688	−0.43778	−0.51491	−0.50392	0.73260	0.26740

l_x_(i): Probability of surviving for index population; l_x_(s): Probability of surviving for standard population.

**Table 9:** Derivation of Smoothed Probability of Surviving and Smoothed Mortality Levels for **Southern** Nigeria

**Age**	**x**	***l***_**x**_**(i)**	**Probabilities of surviving*l***_**S**_**(i)**			**Logit{*l***_***x***_**(*i*)}**	**Logit{*l***_***x***_**(*s*)}**	**Logit{*l***_**x**_**(*l*)**	**Smoothed Probability of Surviving**	**Smoothed Probability of Dying**
			**Level 16**	**Level 17**	**Level 16.21**					
*1*	*2*	*3*	*4*	*5*	*6*	*7*	*8*	*9*	*10*	*11*
15-19	1	0.9231	0.91750	0.92764	0.91963	−1.24262	−1.21866	−1.23807	0.92245	0.07755
20-24	2	0.8940	0.89785	0.91162	0.90074	−1.06613	−1.10275	−1.12216	0.90416	0.09584
25-29	3	0.8910	0.88526	0.90101	0.88857	−1.05050	−1.03810	−1.05751	0.89235	0.10765
30-34	5	0.8893	0.86818	0.88633	0.87199	−1.04181	−0.95934	−0.97875	0.87626	0.12374
35-39	10	0.8683	0.84751	0.86868	0.85196	−0.94301	−0.87501	−0.89442	0.85679	0.14321
40-44	15	0.8705	0.83592	0.85853	0.84067	−0.95269	−0.83160	−0.85101	0.84580	0.15420
45-49	20	0.8338	0.82169	0.84564	0.82672	−0.80640	−0.78128	−0.80069	0.83221	0.16779

l_x_(i): Probability of surviving for index population; l_x_(s): Probability of surviving for standard population.

**Figure 1:**
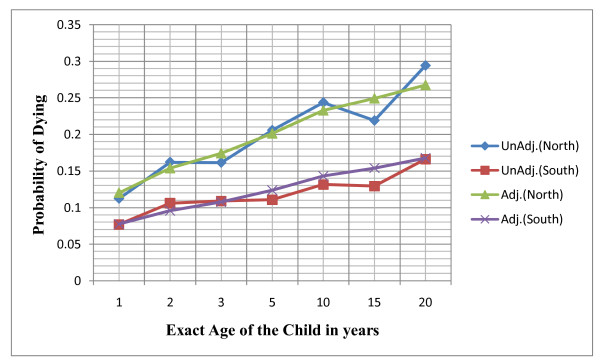
Unadjusted and Adjusted Estimate of Childhood Probabilities of Dying in the Northern and Southern Nigeria: NDHS, 2008.

Figure 1 depicts the results of unadjusted and adjusted childhood mortality probabilities in the northern and southern Nigeria. The data show that clear differences existed between the unadjusted and adjusted childhood probabilities of dying in both regions. Both the unadjusted and adjusted probabilities of dying at all childhood ages were consistently higher among women in the North than their counterparts in the South. Also the probabilities of dying increased steadily with the age of the child.

## Discussion

In this study, the selected socio-demographic characteristics (age, residence, education, wealth index and religion) were significantly associated with region. The subgroup analysis revealed that the women also exhibited regional differences in these characteristics. The study also discovered that across the two regions, majority of the respondents were between the ages of 25–29 and higher number of respondents were living in the rural than urban areas. This finding is in accordance with the latest census report in Nigeria [7]. However, the proportion of rural–urban residents’ women was higher in the north than the south. This is also expected as the geographical distribution of proportion of rural–urban areas in Nigeria show that it was higher in the north than the south [7].

Majority of the women in the northern region had no education, whereas in the south majority had secondary education. The possible reason for this differential is early marriage among female children which is commonly practiced in the north [3,7]. With respect to wealth index, higher proportion of women in the north than the south belonged to poorest wealth index, whereas, majority of the women in the south were in the richest wealth index category. Due to culture and religion, most women in the north, particularly women of childbearing age do not participate actively in labor force. However, in the south, the literacy level had undermined most of the cultural attitudes against women, particularly; those that made women to remain full housewife have accorded women opportunity to engage in one job or the other. Islam was the mostly practiced religion in the north among the women, while in the south it was Christianity. This is expected as residents in the northern and southern Nigeria are predominantly Muslims and Christians respectively [7].

The study is evidenced that significance association existed between experienced childhood mortality and region. The prevalence of experienced childhood mortality in Nigeria was 40.3% and differential existed by region as it was higher in the north (47.3%) than the south (30.9%). Similar patterns of death among children were observed in the northern and southern regions across the selected socio-demographic characteristics, but variation existed in these patterns across subgroups of women in the two regions. Women in the north experienced higher childhood mortality than the south. Poor and harsh climatic conditions in the north and high level of literacy in the south [3] can be attributed to the difference. Better access and utilization of modern health facilities in terms of antenatal care, delivery place and assistance at delivery by women in the South than the North can also be possible explanations for the north–south gap in childhood mortality [3].

Across all age groups, higher childhood deaths experienced was recorded by women in the North than their counterparts in the South. In each of the age group, the percentage of women who experienced childhood mortality increases with increase in age of women. This is expected in any setting, since older women tend to bear more children than the younger ones and as a result having more children who are exposed to the risk of dying than the children of younger women. This increases cumulative childhood deaths among older women than the younger ones [21].

The place of residence is usually seen as a place where differentials in childhood mortality can be observed. The differentials are largely attributed to such factors as the differences in the standards of living, accessibility of public health and medical health care facilities and differences in the social and economic status of families. In either of the regions, rural women experienced higher childhood mortality than urban women. This may not be far from the fact that modern health care utilization viz; affordability, accessibility and acceptability are more common among women in the urban than rural areas [3]. Also high literacy level and access to health information in the urban than rural areas can undermine cultural factors that tend to influence child’s health seeking behaviors. The result is consistent with the findings from the study conducted by Narayan Sastry, 2009. This paper presents an analysis of differentials in child survival by rural–urban place of residence in Brazil and found that Child mortality rates were substantially and significantly lower in urban areas of Brazil. The author's results suggest, however, that the urban advantage does not simply reflect underlying differences in socioeconomic and behavioral characteristics at the individual and household levels; rather, community variables appear to play an independent and important role [22].

The proportion of mothers that have lost at least a child in the past fell consistently as the levels of education increases but at pal in the two regions. This finding is consistent with that reported by Adetunji in 2002 [13,20]. This is an indication that irrespective of the region, education is a fundamental factor to consider in terms of child survival. It erodes socio-cultural ideology of women in terms of providing adequate health needs and care for their children, hence enhancing their survival. The study by Nwogu and his collegues in 2008, showed that the literacy rate and domestic spending on healthcare were statistically significant identified factors influencing childhood mortality in Nigeria and recommended that the country needs a unified approach to healthcare delivery so as to overcome cultural and political divisions in Nigeria [8]. The study by Kirosa and Hogan also supported the findings from the current study. Their study utilized 1994 Population Census of Ethiopia and child mortality levels and trends were estimated using indirect methods, found that enormous variations exist in child mortality by parental education and was highest among children born to illiterate parents [20].

Striking differential existed between childhood mortality experience by mothers who belong to Islamic religious sect in the North and in the South. The proportion of Muslim women in the North who experienced childhood mortality was almost twice that of the South. Possible explanation is that women in the South are more educated than that of the North [7]. Women in the North are predominantly Muslims and bear more children, live shorter births interval, less utilized modern health facilities than their counterparts in the South [7]. The Christian mothers in the South had higher childhood mortality experience than their Muslim counterparts, but reverse is the case for Christians and Muslims in the North. Meanwhile, children of traditional followers in the North had lower childhood mortality than their counterparts in the South.

Wealth index also showed an inverse relationship with childhood death in the two regions. The gap in childhood mortality between the poorest and richest was wider in the North than the South. This is consistent with the study of Kembo and Van Ginneken [23]. It is alarming to know that the percentage of mothers who had experienced childhood mortality in the poorest wealth category in the South was lower than that of the richer in the North. Polygamy and attitudes of heads of the families towards childcare in these regions could be the reasons for the gap. Sharing of income and wealth between members of the family can have adverse effects on every member if the family is large as always found in majority of Northern homes [24].

The study further revealed that there was no significant difference in the risk of childhood mortality in the urban and rural areas in the North however; the difference was significant in the South with rural women more likely to have experienced child deaths than urban women. Children of the poor are significantly more likely to die than that of the rich and the higher the level of education the lower the risk of mortality among children in the two regions.

The probabilities of dying increased with the age of children in the two regions. Across all ages of the children, probabilities of dying were consistently higher in the North than the South. The life-table mortality levels were lower in the North than the South, showing an indication of higher previous childhood mortality experience in the North than in the South. The smoothening of mortality probabilities either reduces or increases the unadjusted mortality probabilities in the two regions. But clear differences existed between the unadjusted and adjusted childhood probabilities of dying in both regions. A similar study conducted in Bangladesh in 1997 supported the findings [25]. The implication of this finding is that better policies are made based on the adjusted estimates, as these will provide true childhood mortality situations in the two regions than those first thought. The adjusted estimates also give a better basis for comparison between nations.

## Conclusions

Childhood mortality probabilities are high in Nigeria and Northern women experienced higher childhood mortality than their counterparts in the South. Adjusted mortality probabilities showed clear deviation from the unadjusted mortality probabilities. Therefore, using the estimated mortality probabilities without refining it in some way can pose serious danger in terms of health and childhood mortality reduction strategies in Nigeria. High childhood probabilities of deaths underscore the need for intervention programmes in Nigeria. Improving women education, particularly in the North may alleviate childhood mortality in Nigeria. Policy makers should also take into consideration of factors such as maternal age, residential area, religion and wealth index while designing programmes to reduce childhood mortality in Nigeria. The need for further studies on childhood mortality in Nigeria is important, particularly in the North where higher childhood mortality was experienced.

## Competing interests

The authors declare that they have no competing interests.

## Authors' contributions

ASA, YOB and FFA contributed to the study protocol. ASA conceived the idea, write the method section, did the data extraction, analysis and interpretation. YOB reviewed the data analysis and wrote the discussion. FFA reviewed relevant literatures. All the authors reviewed and approved the final draft of the manuscript.

## Pre-publication history

The pre-publication history for this paper can be accessed here:

http://www.biomedcentral.com/1471-2458/12/430/prepub
